# A mixed-methods study on the effectiveness and optimization needs of a prescription review training program: from the KAP perspective of returning trainees

**DOI:** 10.1186/s12909-026-10186-x

**Published:** 2026-08-18

**Authors:** Xiangfen Shi, Zhao Yin, Xiaoxu Chen, Xiaoyue Bao, Xiaojing Lu

**Affiliations:** 1 Department of Pharmacy, The First Affiliated Hospital of Zhengzhou University, Zhengzhou, 450052 China; 2 Institute for Hospital Management of Henan Province, Zhengzhou, 450052 China; 3 Henan Drug Clinical Comprehensive Evaluation Center, Zhengzhou, 450052 China

**Keywords:** China, Prescription review, Pharmacy education, KAP model, Mixed-methods, Program evaluation

## Abstract

**Background:**

Prescription review training programs are widely implemented in China to meet increasingly stringent policy requirements for rational drug use. However, evidence on the effectiveness of these programs and the specific needs for their optimization, particularly from the knowledge, attitude, and practice (KAP) perspective of returning pharmacists, remains limited. This gap constrains efforts to refine training systems and support the implementation of national pharmaceutical policies.

**Methods:**

An exploratory sequential mixed-methods design grounded in the KAP model was employed. Phase I involved semi-structured interviews with 15 returning trainees, with thematic analysis adopted for data coding and thematising; Phase II administered a KAP questionnaire to trainees, quantitative data were analyzed using descriptive statistics, Pearson correlation, and binary logistic regression.

**Results:**

Qualitative interview revealed that trainees reported good knowledge acquisition and positive attitude transformation, but also faced practice barriers (limited pre‑review platform support, physician communication difficulties, and specialty misalignment). Quantitative survey of 394 trainees showed mean knowledge scores of 32.6±3.2, attitude scores of 28.7±2.4, and practice scores of 32.3±3.6, with significant positive correlations among them. Regression analysis identified low usefulness of role‑playing and insufficient communication training demand were independent associated factors for poor practice translation (P<0.05), while workplace contextual factors(e.g. pre‑review platform) were not significant. Both qualitative and quantitative findings consistently indicated strong trainee demand for extended training, specialty focused content, communication practice, and post-training support.

**Conclusion:**

Prescription review training effectively improves pharmacists’ knowledge and professional attitude. However, despite high knowledge and practice scores, qualitative findings revealed persistent self reported difficulties in applying training to practice, indicating a discrepancy between measured outcomes and perceived challenges. The two finding are complementary rather than contradictory. Addressing this discrepancy requires integrated interventions, including strengthened communication skills training, specialty-adapted content, improved institutional support platforms, and sustainable post-training follow-up.

**Supplementary Information:**

The online version contains supplementary material available at https://doi.org/10.1186/s12909-026-10186-x.

## Introduction

The role of the pharmacist has been progressively redefined within China’s healthcare system, evolving from traditional dispensing to assuming critical responsibility for prescription review and rational drug use [1, 2]. This transformation has been driven by a systematic and evolving national policy framework. Initial mandates were established with the 2007 “Prescription Management Measures” and the 2011 “Regulations of pharmaceutical administration in medical institutions”, which laid the foundational principle that pharmacists must review prescription appropriateness [3, 4]. A significant shift occurred in 2018 with the “Medical Institution Prescription Review Standards”, which explicitly designated the pharmacist as the “first responsible person” for review, marking a significant advancement in professional accountability [5]. Subsequent policies, including the 2020 “Opinions on Strengthening Pharmaceutical Management” and the 2025 “Informatization Guidelines for Compact County Healthcare Communities,” have further reinforced this role and expanded the scope of review to encompass integrated healthcare systems [6, 7].As a result, prescription review has become a mandatory daily duty for pharmacists who perform prescription dispensing and review in medical institutions.Against this policy backdrop, the effective translation of pharmacists’ post-training review competencies into clinical practice has become a key link to ensure policy implementation and improve medication safety.

To support the implementation of these policies and strengthen pharmacists’ competency in prescription review, health administrative authorities at both national and local levels have launched specialized training programs. For instance, the National Health Commission’s “Shortage Talent Pharmacist Post Training Program”, a key initiative supported by the 2024 central government budget for healthcare talent, is a voluntary program with institutional encouragement (no fees charged to participants; a certificate is awarded upon passing the final examination).It specifically cultivates personnel in three core training modules: prescription review and dispensing, pharmacist-led patient consultations, and inpatient medication monitoring [8]. Meanwhile, provincial health commissions have organized “Primary-Level Pharmacist Prescription Review Capacity Training” courses, focusing on relevant laws and regulations, key medication principles across disciplines, and practical case analyses, thereby building systematic capacity among pharmacists in primary care settings [9, 10]. However, evaluations of most existing training programs in China have primarily focused on assessing short-term, direct training outcomes, such as course completion rates, satisfaction surveys, and immediate skill assessments [11, 12]. Several international studies have similarly evaluated medication review or prescription review training programs using short‑term indicators, including a Norwegian work‑integrated training program for hospital clinical pharmacists [13], a Japanese randomized controlled trial on online team‑based learning for clinical medication reviews in older adults [14], and a Finnish competence assessment study for medication review skills after an advanced internship [15]. Despite these efforts, a common limitation across both domestic and international studies is the lack of follow‑up assessment on whether trainees actually translate their acquired competencies into routine clinical practice after returning to work. This approach pays insufficient attention to the sustainability of learning and its translation into workplace practice after trainees return to their posts. Consequently, many trainees who have returned to their clinical posts after completing the training report difficulties in applying the competencies demonstrated during training to real-world clinical settings.

The Knowledge, Attitude, and Practice (KAP) model provides a robust theoretical framework for evaluating the effectiveness of health behavior interventions [16]. It systematically explains the interrelationships among knowledge acquisition, attitude formation, and behavioral practice, thereby offering a scientific basis for multidimensional assessment [17]. To date, no study has adopted the KAP model as a core analytical framework to systematically investigate the full-chain training effects (knowledge-acquisition, attitude-formation, practice-implementation) of participants in national and provincial prescription review training programs. Moreover, there has been limited in-depth exploration of the specific implementation barriers these trainees encounter in actual work settings. As direct stakeholders in the training process, feedback from returning trainees can not only reflect the relevance of training content to their post-training roles but also identify key factors that facilitate or hinder the application of learned competencies. The absence of this perspective in current evaluations makes it difficult to assess the true impact of training programs or to generate empirical evidence for systemic improvement.

To address these research gaps, this study adopts the KAP model as its theoretical foundation and employs an exploratory sequential mixed-methods design. The specific objectives are to: 1)evaluate the effectiveness of the prescription review training program in terms of knowledge, attitude, and practice; 2) identify the key barriers to practice translation from the perspective of returning trainees; 3) determine the independent factors associated with poor practice translation; and 4) propose actionable strategies for training optimization (e.g., strengthening communication skills and improving post-training support mechanisms). The findings are expected to provide an empirical basis for developing a more practice-oriented and needs-aligned prescription review training system, ultimately contributing to improved quality of clinical prescription review and enhanced patient medication safety.

## Methods

### Design

This study employed an exploratory sequential mixed-methods design [18, 19]. This design consists of an initial qualitative phase followed by a quantitative phase, in which findings from the qualitative phase inform the development of the quantitative instrument, thereby enhancing content validity for pharmacy training evaluation.

#### Description of the prescription review training program

The evaluated program was mainly a one‑month standard training (388/394, 98.5%). Four trainees attended a three‑month and two a six‑month program; their prescription review content was identical, only with supplementary modules such as pharmacy clinics added.

The one month training was full time, combining in‑person lectures with hands on practice. Lectures covered over 30 topics, including prescription review standards, rational use of antimicrobials, antineoplastics and high-alert medications, pediatric medication, cancer pain management, adverse reaction mining, and rule establishment. During practice sessions, trainees performed outpatient, emergency and inpatient medication reviews, and report writing under the guidance of certified pharmacists who had completed prescription review training and possessed substantial practical experience. Upon completion, a standardized written and practical examination organized by the provincial health commission was mandatory for certification. Participation was voluntary but encouraged by employers, with all training fees covered by provincial health budgets.

### Phase Ⅰ: qualitative study

#### Data collection and sampling

**Fig. 1:**
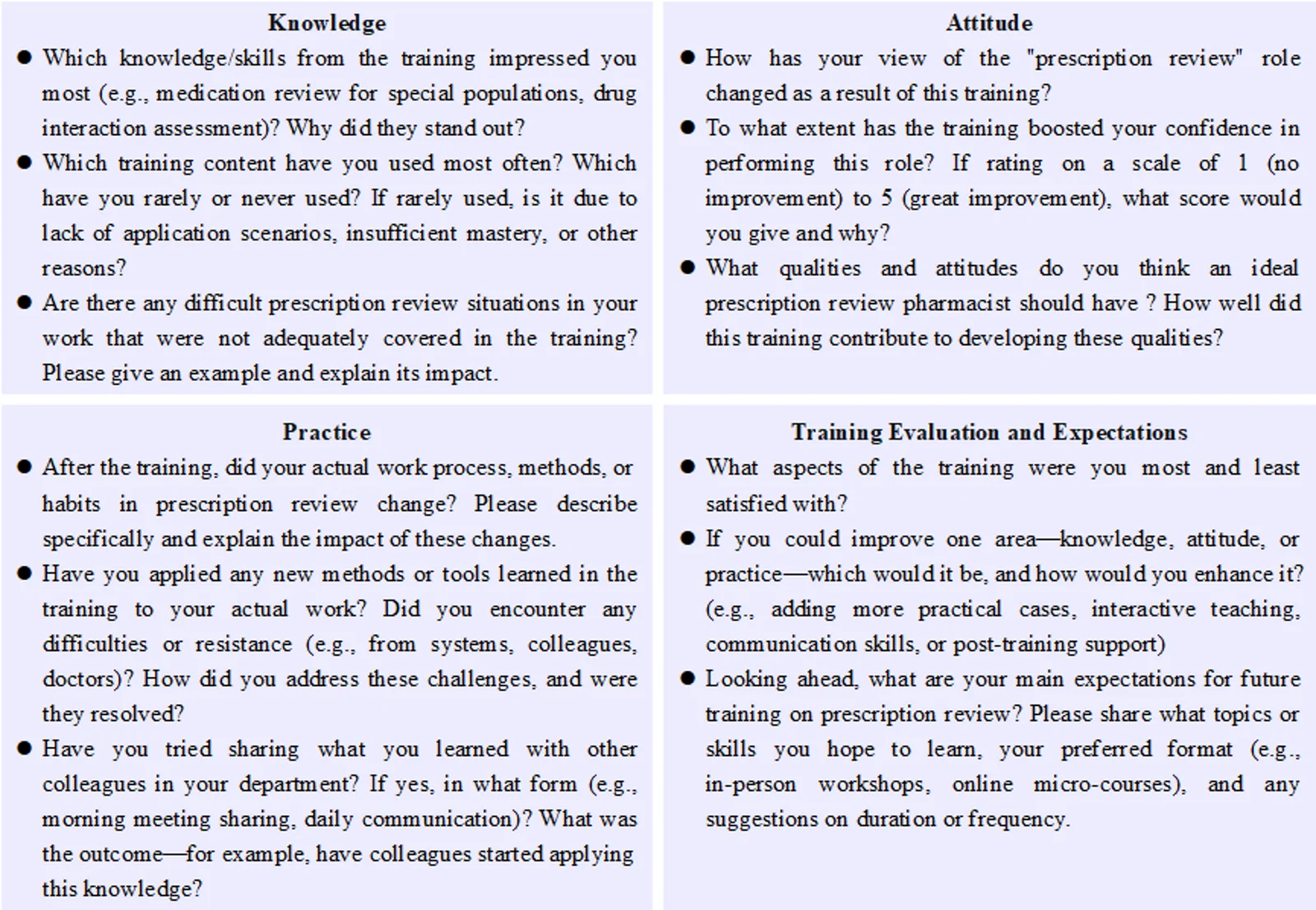
Interview guide grounded in the KAP theory

A semi-structured interview guide was developed based on the KAP model to systematically explore the post-training experiences of returning trainees. The guide was designed to elicit detailed insights into the applicability of training content, perceived practice challenges, changes in professional practice and training optimization needs. The interview guide grounded in the KAP theory is presented in Fig. 1.

Data were collected through in-depth, one-on-one, semi-structured interviews conducted via telephone. Eligible participants met the following criteria: (i) completed a national or provincial prescription review training program and obtained the formal certificate; (ii) returned to their clinical posts and engaged in prescription review duties; and (iii) voluntarily consented to take part in the study and the interview.

We used a stratified purposive sampling approach based on hospital grade, professional title, and years of practice. Within each stratum, we used maximum variation sampling to select potential participants. Potential participants who met the inclusion criteria were contacted via telephone. Two researchers independently coded each interview; recruitment stopped after three consecutive interviews yielded no new codes (confirmed by consensus) [20].

Following this criterion, saturation was achieved after 15 interviews. During the recruitment process, a total of 20 eligible trainees were approached, of whom 15 participated (5 declined). Interviews were conducted between January 25 and February 2, 2026, at prearranged times by a principal researcher (XFS), with another researcher (XJL) assisting with recording, and without external interruptions. Each interview lasted 30 ~ 45 min.

#### Data analysis

Interview data were analyzed using a thematic analysis approach, a method widely employed in qualitative research to identify, analyze, and report patterns within data. We adopted the six-phase procedural framework proposed by Braun and Clarke for the analysis [21]. Interviews were transcribed and verified. Coding was guided by the KAP framework, while also allowing new themes (e.g., training expectations) to emerge from the data. While the KAP model provided a deductive coding structure, we also used inductive analysis to capture context-related factors that were repeatedly mentioned by participants, such as workplace constraints affecting practice translation. For the qualitative component, we defined “knowledge” as learning points from training, “attitude” as changes in professional identity and confidence, and “practice” as reported behaviors and barriers in real work. Two researchers coded independently; codes were then grouped into candidate themes and refined through iterative discussion. Disagreements were resolved by discussion or by consulting a senior expert, and themes were finalized with a senior expert. No formal inter-coder reliability statistic was calculated, as consensus was reached iteratively. NVivo12 software was used to manage and analyze the data. The final report was prepared in accordance with the Consolidated Criteria for Reporting Qualitative Studies (COREQ) checklist [22], which is presented in Table S1 (supplementary material).

### Phase Ⅱ: quantitative study

#### Questionnaire development and final instrument

Based on core themes discerned in the qualitative phase, a structured KAP questionnaire was developed. For example, the theme “Major Barriers in Practice” informed items on physician communication difficulties and workplace constraints, while “Optimization Suggestions” generated items on role‑playing helpfulness and demand for post‑training support. The initial items were derived from qualitative findings and reviewed by a panel of five experts (two clinical pharmacy professors, two senior pharmacy directors, and one methodology specialist) for relevance and clarity. A two-round Delphi expert consultation was conducted with 17 experts. Experts rated the importance of each item on a 5-point Likert scale(1 = not important, 5 = very important). The item-level Content Validity Index (I-CVI) and scale-level average CVI (S-CVI/Ave) were calculated. Following the method described by Lynn, the I-CVI was calculated as the proportion of experts rating an item as 4 or 5 on the 5-point scale [23]. Items with I-CVI ≥ 0.78 were retained, as recommended by Polit [24]. The S-CVI/Ave was calculated as the average of all I-CVI values [25]. Items with I-CVI below 0.78 were revised or removed. In the first round, all items achieved I‑CVI ≥ 0.78 (range 0.79–0.89, S‑CVI/Ave = 0.87). Based on feedback from two experts, minor wording adjustments were made. A second round was conducted to confirm consensus, and no further changes were needed. The refined questionnaire was then pilot-tested with 15 pharmacists from the target population (who had completed similar training but were not involved in the main study) to assess face validity, including clarity, wording, and completion time. Minor wording adjustments were made based on their feedback. The pilot study (*n* = 15) showed acceptable to good internal consistency: Cronbach’s α was 0.96 for the total scale, 0.88 for the knowledge domain, 0.93 for the attitude domain, and 0.92 for the practice domain. The questionnaire developed specifically for this study is available as a supplementary file (see Supplementary Questionnaire).

The final questionnaire consisted of five sections: a demographic and professional background section; a knowledge scale (7 items) assessing perceived learning outcomes; an attitude scale (6 items) measuring changes in professional confidence and role perception; a practice scale (7 items) evaluating the application of training, including changes in workflow, utilization frequency of learned skills, and encountered barriers; and a training evaluation and expectations Sect.  (23 items) focusing on overall feedback, suggestions for improvement, and preferences for future training. KAP domains used 5-point Likert scales (0 = strongly disagree to 5 = strongly agree). The training evaluation and expectations section used the same scale (0 = not at all useful/needed to 5 = extremely useful/necessary).

Notably, factor analysis was not performed to verify the construct validity of the three KAP domains, as the scale structure was established based on the classic theoretical KAP framework and further supported by expert review and pilot testing.

#### Data collection and sampling

A cross-sectional online survey was administered to pharmacists in Henan Province who had completed prescription review training and returned to work. A convenience sampling method was used. The questionnaire was distributed via Questionnaire Star (Changsha Ranxing Information Technology Co., Ltd.) over a one-week period through provincial training base coordinators. The coordinators posted a quick response (QR) code link in the official work WeChat groups of eligible trainees. No reminders were sent, and individual completion was not tracked. Prior to the survey, an electronic information page detailing the study purpose, procedures, and data anonymity was presented. All participants were required to actively indicate their consent by selecting an option that stated, “I have read and understood the above information and voluntarily agree to participate in this study” before they could proceed to the questionnaire items.

#### Quantitative data analysis

Sample size was estimated using the events per variable (EPV) rule for logistic regression. Based on the pilot study, we assumed a 25% event rate for poor practice translation. With up to 10 predictor variables, the minimum required sample size was calculated as (10 × 10) / 0.25 = 400. The final sample of 394 closely approached this target.

Data from the survey were analyzed using SPSS 26.0 software. Descriptive statistics (frequencies, means, and standard deviations) were used to summarize the demographic characteristics of the respondents and their KAP scores; Pearson correlation analysis was used to explore the correlations among knowledge, attitude and practice scores; Binary logistic regression analysis was conducted to identify independent associated predictors of poor practice translation (dependent variable: 1 = practice score < 30, 0 = ≥ 30). This cutoff was justified by the pilot study (*n* = 15), in which 26.7% of participants scored below 30 (the 26th percentile). Variables were selected based on theoretical and clinical relevance to pharmacy practice translation, including dichotomous variables (hospital prescription pre-review platform, cross-department rotation during training, perceived helpfulness of role-playing, demand for communication skills training; all coded as 0 = no/negative, 1 = yes/positive) and continuous variables (age, years of pharmacy work, years of prescription review experience, months since returning to work). In addition, knowledge and attitude scores were forced into the model based on the KAP framework. The variable “expectation for post-training mentor guidance” was initially considered but excluded from the final model because only eight participants (2.0%) responded negatively and all of them had poor practice scores (< 30), indicating complete separation that prevents maximum likelihood estimation from converging. Training duration was excluded because 98.5% of participants had the same duration (one month), resulting in insufficient variability. The Enter method was used for variable entry. Multicollinearity was assessed using variance inflation factors (VIF); most VIF values were below 5, with age (6.161) and years of pharmacy work (6.403) showing moderate multicollinearity as expected, and no VIF exceeded 10. Model fit was evaluated using the Hosmer-Lemeshow test (χ²=5.649, df = 7, *p* = 0.581) and Nagelkerke R²=0.692, indicating good model fit. Odds ratios (ORs) and 95% confidence intervals (CIs) were calculated to assess associations. A two-sided *P* < 0.05 was considered statistically significant for all analyses.

### Ethical consideration

Ethical approval for the study was obtained from the First Affiliated Hospital of Zhengzhou University Institutional Review Board (No.2026-KY-0053). Written informed consent was obtained from all the study participants.

### Trustworthiness

We took measures to ensure the trustworthiness of this study as described in our previous study [20, 26]. These measures are outlined below: (1) the research team included members with experience in conducting mixed-method, qualitative, or quantitative studies, and the principal investigator maintained close contact with methodological experts to continuously address difficulties in the research process; (2) To enhance credibility and confirmability, we employed triangulation through diverse participant recruitment (different hospital grades, professional titles, and years of practice) and independent dual coding by two researchers, with disagreements resolved through consensus or consultation with a senior expert. (3) An audit trail was maintained, documenting all coding decisions, thematic revisions, and analytical choices.4) Peer debriefing was conducted through regular team meetings where emerging themes were reviewed and discussed critically by other team members. 5) The principal researcher maintained reflexivity by consciously reflecting on potential interpretive bias during analysis, and discussing emerging themes with other team members to ensure neutrality. 6) In addition, qualitative findings were confirmed by participants post-analysis to verify consistency and authenticity, and the whole study was conducted in strict accordance with standardized operational procedures.

## Results

### Phase I: qualitative findings from interviews

#### Participant characteristics

A total of 15 participants were enrolled in the interviews. Demographically, the group consisted of 9 females and 6 males, with a mean age of 35.73 ± 5.20years, and all held a bachelor’s degree or above. The sample exhibited diversity across key professional dimensions: in terms of hospital level, 6 participants were from Grade III hospitals and 9 from Grade II hospitals; regarding professional title, the cohort comprised 6 Pharmacists, 8 Chief Pharmacists, and 1 Associate Chief Pharmacist. In relation to training and experience, all participants had completed a prescription review training program, with the most recent training session occurring on average 1.79 ± 0.65 months prior to the interviews. Professionally, they had an average of 11.80 ± 5.02 years of experience in pharmaceutical practice, including 0.87 ± 0.72 years specifically dedicated to prescription review.

#### Major themes and sub-themes

Thematic analysis of the interview transcripts revealed four core themes that directly correspond to the KAP evaluation framework and the training feedback dimension: (1) Knowledge Acquisition and Application Gaps, (2) Attitudinal and Perceptual Shifts, (3) Behavioral Changes and Practice Barriers, and (4) Training Evaluation and Future Expectations. Each theme encompasses several salient sub-themes, detailed in Table S2.

The analysis indicates that the training achieved significant results at the Knowledge (K) and Attitude (A) levels. Trainees acquired advanced and practical review knowledge; one participant noted, “What left the deepest impression was the rational clinical use of anti-tumor drugs” (T4). They also underwent a transformation in role perception from passive dispensers to active safety “gatekeepers,” with a substantial boost in self-confidence. As another trainee stated, “Pharmacists are the first responsible persons for medication safety” (T5). However, at the practice level, a noticeable “knowledge-attitude-practice” gap emerged. Although trainees’ review behaviors became more rigorous and they attempted to introduce new methods, contextual barriers such as platform deficiencies, resistance in clinical communication, and specialty misalignment severely constrained the effective translation of knowledge into practice. One trainee explained, “Communication skills really need practice—especially when talking with physicians” (T9). Trainees’ evaluations and expectations further suggest clear directions for optimization: they highly recognized the teaching quality but were dissatisfied with the rushed schedule and logistical issues. Their expectations centered on longer training duration, more specialty specific content, more hands on and communication training, and a sustainable post training platform to better bridge knowledge and practice, with one trainee hoping, “Learning shouldn’t just end after one month” (T9).

### Phase II: preliminary quantitative results from the survey

#### Sample characteristics and response

A total of 396 trainees submitted the questionnaire; 394 provided complete data (two were excluded due to incomplete responses). Detailed demographic and practice characteristics are summarized in Table 1.

**Table 1:** Demographic characteristics and response of returning trainees (*n* = 394)

Characteristics	Category/Description	Number	Percentage/Statistic
Gender	Male	104	26.4%
	Female	290	73.6%
Age (years)	Mean ± SD	-	36.0 ± 5.8
Educational level	Associate Degree	20	5.1%
	Bachelor’s Degree	320	81.2%
	Master’s Degree	54	13.7%
Title	Junior-level Titles	358	90.9%
	Intermediate-level Titles	34	8.6%
	Senior-level Titles	2	0.5%
Working years (years)	Mean ± SD	-	12.3 ± 6.4
Years of experience in prescription review (years)	Mean ± SD	-	5.5 ± 5.9
Training Duration for Prescription Review Positions	1 month	388	98.5%
	3 months	4	1.0%
	6 months	2	0.5%
Did you rotate to other departments during the training period?	Yes	212	53.8%
	No	182	46.2%
Time since returning to work after training (months)	Mean ± SD	-	5.1 ± 6.5
Hospital grade	Grade III	226	57.4%
	Grade II	156	39.6%
	Community Health Service Center/Station	6	1.5%
	Township Hospital	6	1.5%
Has your hospital implemented a prescription pre-review platform?	Yes	202	51.3%
	No	192	48.7%
Main position after return	Prescription Review	284	72.1%
	Dispensing and Prescription Review	108	27.4%
	Clinical Pharmacy and Prescription Review	2	0.5%

#### Responses to knowledge, attitude, and practice statements

The internal consistency of the questionnaire was assessed using Cronbach’s α. For the full sample (*n* = 394), the total scale α was 0.969, and the subscale α values for knowledge, attitude, and practice were 0.919, 0.964, and 0.950, respectively, indicating excellent reliability. According to provincial training records, approximately 700 pharmacists completed the training during the study period; our sample of 394 therefore covers over 50% of this population. However, exact population data for formal representativeness testing were not available.

The mean (± SD) knowledge, attitude, and practice scores were 32.6 ± 3.2, 28.7 ± 2.4, and 32.3 ± 3.6, respectively (Table 2). Participants demonstrated high levels of knowledge and positive attitude, and the practice scores were numerically similar to the knowledge scores. Pearson correlation analysis (Table S3) showed significant positive correlations between knowledge and attitude (*r* = 0.781, 95% CI [0.745–0.812]), knowledge and practice (*r* = 0.821, 95% CI [0.791–0.846]), and attitude and practice (*r* = 0.708, 95% CI [0.658–0.750]), all *P* < 0.05. The scatterplots (Supplementary Figure S1) show that the relationships between variables are roughly linear and that the data points are evenly spread around the regression lines, meeting the basic requirements for Pearson correlation.

**Table 2:** Mean knowledge, attitude, and practice scores (*n* = 394)

Variable	Mean	SD	Minimum	Maximum	Full Score
Knowledge score	32.6	3.2	21	35	35
Attitude score	28.7	2.4	18	30	30
Practice score	32.3	3.6	20	35	35

Binary logistic regression analysis results are presented in Table 3. Perceived helpfulness of role-playing during training was a strong associated factor (OR = 0.104, 95%CI:0.019–0.560, *P* < 0.001), meaning participants who found role-playing helpful were approximately 9.6 times more likely to have good practice translation compared with those who did not. Additionally, perceived demand for communication skills training was a significant associated factor (OR = 0.142, 95%CI:0.030–0.669, *P* = 0.014), indicating that trainees who recognized the importance of communication skills were approximately 7.0 times more likely to have good practice translation. Knowledge score was also a significant independent predictor (OR = 0.475, 95% CI: 0.373–0.606, *p* < 0.001), while attitude score was not significant (*p* = 0.987). Age, years of pharmacy work experience, years of prescription review experience, hospital prescription pre-review platform implementation, cross-department rotation during training, and time since returning to work were not independent influencing factors (all *P* > 0.05).

**Table 3:** Binary logistic regression results for associated factors of poor practice translation (*n* = 394)

Variable	B	SE	Wald χ²	*P*-value	OR	95% CI for OR (Lower, Upper)
Age	0.006	0.069	0.008	0.930	1.006	0.879,1.151
Years of pharmacy work	0.026	0.062	0.177	0.674	1.027	0.909,1.160
Years of prescription review experience	-0.031	0.036	0.768	0.381	0.969	0.904,1.039
Months since returning to work	0.007	0.022	0.101	0.750	1.007	0.965,1.051
Hospital prescription pre-review platform	0.023	0.372	0.004	0.951	1.023	0.493,2.122
Cross-department rotation during training	-0.192	0.325	0.350	0.554	0.825	0.436,1.561
Perceived helpfulness of role-playing	-2.260	0.857	6.945	0.008	0.104	0.019,0.560
Perceived demand for communication skills training	-1.950	0.790	6.095	0.014	0.142	0.030,0.669
Knowledge	-0.744	0.124	36.032	0.000	0.475	0.373,0.606
Attitude	-16.341	1036.540	0.000	0.987	0.000	0.000,-
Constant	516.836	31096.187	0.000	0.987	2.877E + 224	

Poor practice translation was defined as practice score < 30 (coded as 1), and good practice translation as score ≥ 30 (coded as 0)

*OR *odds ratio, *CI* confidence interval

Returning trainees’ responses to the KAP statements are summarized in Table S4. Regarding the knowledge dimension, an overwhelming majority of participants agreed that the training positively impacted their professional knowledge reserve. Specifically, 98.5% of respondents agreed the training comprehensively covered core review knowledge, 98.0% found it highly relevant to their work needs, and 98.5% reported significant improvement in their professional knowledge level.

For the attitude dimension, nearly all respondents reported enhanced perceptions and beliefs: 99.5% gained a deeper understanding of the role in patient safety and a strengthened sense of responsibility as a “gatekeeper of medication safety”, 99.0% reported increased confidence in performing review work well, 98.5% indicated greater willingness to proactively participate in rational medication interventions.

Concerning the practice dimension, most participants reported positive changes in work behavior: 94.4% had applied the learned standard processes to daily work, 97.0% intervened more proactively on risky prescriptions, 91.4% habitually used recommended tools like evidence-based databases, 97.5% actively shared new knowledge with colleagues.

#### Analysis of survey results on teaching evaluation and optimization expectations

Survey results indicated strong trainee approval of the current training and clear expectations for further improvement, consistent with the practice barriers and qualitative findings (Table S5). Most teaching methods and topics received high approval ratings (≥ 95%), reflecting broad satisfaction. Notably, trainees expressed particularly high demand (≥ 97%) for post‑training support such as communication platforms, mentorship, and continuous learning resources, indicating that one time training was insufficient to overcome long term practice barriers. Overall, the training framework was well aligned with trainee needs, and future improvements should focus on strengthening key content and establishing sustainable long term support to enhance both immediate and continuous professional development.

## Discussion

### Principal findings

This mixed methods study, based on the KAP framework, evaluated the effectiveness of prescription review training programs and explored factors underlying the practice implementation gap. Two key findings emerged.

First, the training significantly improved participants’ knowledge and attitude. Quantitative results showed high mean scores for knowledge and attitude, with a strong positive correlation between them, indicating effective transformation from knowledge acquisition to positive professional attitude. Qualitative data further revealed a clear shift in professional identity, from passive dispensers to proactive medication safety gatekeepers, accompanied by substantial improvements in self-confidence. These findings confirm that the program achieved its primary educational objectives [11, 12].

Second, a discrepancy emerged between the quantitative and qualitative findings. Although practice scores were high and strongly correlated with knowledge scores, qualitative interviews revealed that trainees perceived substantial difficulties in applying training content to real-world practice, including platform deficiencies, resistance in clinical communication, and specialty misalignment. Moreover, binary logistic regression showed that platform factors were not independent predictors of poor practice translation; only communication-related factors reached statistical significance. This apparent inconsistency is addressed in detail below.

### The nature and sources of the practice gap

The apparent discrepancy described above can be explained by two reasons. First, qualitative methods capture perceived barriers, while regression identifies predictors requiring variability across participants. Second, workplace contextual factors likely lacked sufficient variability in our sample. In contrast, communication related factors showed greater inter‑individual variability, making them statistically significant. Thus, the two findings are complementary rather than contradictory.Three interrelated barriers to practice implementation were identified in the qualitative analysis.

Workplace contextual factors were a key recurrent theme. Nearly half of the participants worked in hospitals without a prescription pre-review platform. Among those with access to such platforms, poor usability, rigid rule settings, and outdated knowledge bases substantially limited the application of trained competencies. These findings indicate that individual capacity building cannot achieve optimal outcomes without supportive institutional systems.

Interpersonal-communicative barriers represented a second major challenge. Many participants reported persistent difficulties in communicating with physicians, especially when questioning prescribing decisions from senior clinicians. Despite high demand for communication skills training, this domain was underrepresented in the current curriculum.

Specialty-practice misalignment was the third barrier. Pharmacists from specialized hospitals noted that general training content was poorly matched to their institutional formularies and patient populations, which reduced the practical relevance of the training and impeded real world application.

### Implications for training program optimization

Several actionable strategies can be proposed to optimize future training. Given the disconnect between learning and application reported by trainees and the high proportion without a pre-review platform, better alignment between training content and workplace conditions, together with institutional investment in pre-review platform, is essential to address these reported difficulties in practice application. As trainees requested longer and more specialty specific training, extending training duration and restructuring the curriculum into modular courses may allow more in-depth practice and specialty-specific learning. Given that communication related variables were the only significant correlates in the regression, strengthening structured communication skills training, including expanded and supervised role-playing exercises, is urgently needed. Supported by the > 97% demand for post‑training support, establishing a sustainable post-training support framework, such as mentorship, peer communication platforms, and updated case libraries, would help maintain long term competency improvement.

### Limitations

This study has several limitations that should be considered. First, the cross-sectional design captured self-reported practice at a single time point (mean 5.1 months post-training); thus, long-term dynamic changes in practice could not be assessed. Second, the sample has limited generalizability in terms of region and professional hierarchy. All participants were recruited from Henan Province, so the finding may not apply to regions with different healthcare resources and training systems. Furthermore, given the distinct healthcare policies, pharmacist scope of practice, and training systems in China, the results may not be directly transferable to other countries with different institutional and regulatory contexts. Future cross-national studies are needed to explore the applicability of these findings in diverse settings. The sample was also disproportionately dominated by junior pharmacists (90.9%) and tertiary hospital staff (57.4%), with scarce representation of senior pharmacists and primary care practitioners. Although this distribution reflects the actual attendance of the training program, it limits the extrapolation of the results. In addition, small subgroup sample sizes precluded valid subgroup analyses due to insufficient statistical power. Third, this study has inherent measurement limitations. The high Cronbach’s α values indicated good internal consistency but also suggested potential item redundancy, which may be partly influenced by the large sample size. Furthermore, the high KAP scores may be affected by ceiling effects and social desirability bias, as self-reported assessments tend to overestimate positive practice outcomes. In addition, the attitude variable showed instability in the regression model (extremely large standard error and non-significant p-value), likely due to its high correlation with knowledge (*r* = 0.78). This multicollinearity may have affected the precision of the attitude estimate, and the result should therefore be interpreted with caution. Fourth, the cutoff value used to define poor practice translation was determined arbitrarily based on sample distribution rather than established clinical or methodological standards, which may affect the comparability and robustness of the regression results.

### Future research directions

Based on the above limitations, several directions for future research are proposed. First, longitudinal studies are needed to track changes in trainees’ KAP and practice over time, identify key time points for post-training support, and establish causal relationships between training and practice improvement. Second, the current scale could be optimized by removing redundant items to improve efficiency while maintaining reliability. In addition, combining objective practice indicators with anonymous assessments may help reduce ceiling effects and social desirability bias, leading to more accurate evaluation of training effects. Third, multi-centre and cross-regional studies with balanced sampling are needed to verify the external validity and generalizability of the findings; larger subgroup samples would enable sufficiently powered stratified analyses to explore heterogeneous training effects across different practitioner and institutional backgrounds. Fourth, future studies should adopt standardized clinical cutoffs to enhance the robustness of statistical results.

## Conclusion

This mixed-methods study suggests that a prescription review training program can effectively improve pharmacists’ knowledge and professional attitude. However, while quantitative scores were high and strongly correlated, qualitative findings revealed persistent self reported difficulties in applying training to practice, indicating a discrepancy between measured outcomes and perceived challenges. Qualitative findings highlighted workplace contextual, interpersonal, and specialty-related barriers, while quantitative regression indicated communication related factors as associated factors against poor practice translation.

This discrepancy mainly arises from the fact that workplace contextual and specialty-related barriers were widespread but lacked variability, whereas communication-related factors showed greater variability and thus reached significance. The two findings are complementary rather than contradictory. Therefore, both should be viewed as complementary strategies. Future training should strengthen communication training and institutional workplace contextual support, and implement modular courses to meet specialty needs. Combining both strategies can more effectively translate knowledge into practice and further improve medication safety.

## Supplementary Information

Supplementary Material 1.

## Data Availability

The datasets used during the current study are available from the corresponding author upon reasonable request.
