# Supplementary material for: A mixed-methods study on the effectiveness and optimization needs of a prescription review training program: from the KAP perspective of returning trainees
Source: BMC Med Educ. 2026 Aug 18;26:1689. doi: 10.1186/s12909-026-10186-x (PMC13685306; doi:10.1186/s12909-026-10186-x)
Supplement: Supplementary file 1 [file 12909_2026_10186_MOESM1_ESM.docx]

**Table S1 Consolidated criteria for reporting qualitative studies (COREQ): 32-item checklist**

| No Item | Guide questions/description | Answers |
| --- | --- | --- |
| **Domain 1: Research team and reflexivity** | | |
| Personal Characteristics | | |
| 1. Interviewer/facilitator | Which author/s conducted the interview or focus group? | Xiangfen Shi and Xiaojing Lu |
| 2. Credentials | What were the researcher’s credentials? E.g. PhD, MD | Xiangfen Shi, MS; Xiaojing Lu, MS |
| 3. Occupation | What was their occupation at the time of the study? | Hospital pharmacists |
| 4. Gender | Was the researcher male or female? | Xiangfen Shi, Female; Xiaojing Lu, Female. |
| 5. Experience and training | What experience or training did the researcher have? | Theoretical training and experience of conducting several qualitative studies with other groups. |
| Relationship with participants | | |
| 6. Relationship established | Was a relationship established prior to study commencement? | Yes |
| 7. Participant knowledge of the interviewer | What did the participants know about the researcher? e.g. personal goals, reasons for doing the research | Reasons for doing the research |
| 8. Interviewer characteristics | What characteristics were reported about the interviewer/facilitator? e.g. Bias, assumptions, reasons and interests in the research topic | Reasons and interests in the research topic |
| **Domain 2: study design** | | |
| Theoretical framework | | |
| 9. Methodological orientation and Theory | What methodological orientation was stated to underpin the study? e.g. grounded theory, discourse analysis, ethnography, phenomenology, content analysis | Phenomenology. |
| Participant selection | | |
| 10. Sampling | How were participants selected? e.g. purposive, convenience, consecutive, snowball | Purposive |
| 11. Method of approach | How were participants approached? e.g. face-to-face, telephone, mail, email | face-to-face or video chat. |
| 12. Sample size | How many participants were in the study? | 15 |
| 13. Non-participation | How many people refused to participate or dropped out? Reasons? | a total of 20 eligible trainees were approached, of whom 15 participated (5 declined) |
| Setting | | |
| 14. Setting of data collection | Where was the data collected? e.g. home, clinic, workplace | face-to-face or video chat. |
| 15. Presence of non-participants | Was anyone else present besides the participants and researchers? | No |
| 16. Description of sample | What are the important characteristics of the sample? e.g. demographic data, date | Yes, demographic data. |
| Data collection | | |
| 17. Interview guide | Were questions, prompts, guides provided by the authors? Was it pilot tested? | Yes |
| 18. Repeat interviews | Were repeat interviews carried out? If yes, how many? | No |
| 19. Audio/visual | Did the research use audio or visual recording to collect the data? | Audio recording |
| 20. Field notes | Were field notes made during and/or after the interview or focus group? | Yes |
| 21. Duration | What was the duration of the interviews or focus group? | 25-50 min |
| 22. Data saturation | Was data saturation discussed? | Yes |
| 23. Transcripts returned | Were transcripts returned to participants for comment and/or correction? | Yes |
| **Domain 3: analysis and findingsz** | | |
| Data analysis | | |
| 24. Number of data coders | How many data coders coded the data? | Two |
| 25. Description of the coding tree | Did authors provide a description of the coding tree? | Yes |
| 26. Derivation of themes | Were themes identified in advance or derived from the data? | Derived from the data |
| 27. Software | What software, if applicable, was used to manage the data? | NVIVO 12 |
| 28. Participant checking | Did participants provide feedback on the findings? | Yes |
| Reporting | | |
| 29. Quotations presented | Were participant quotations presented to illustrate the themes / findings? Was each quotation identified? e.g. participant number | Yes |
| 30. Data and findings consistent | Was there consistency between the data presented and the findings? | Yes |
| 31. Clarity of major themes | Were major themes clearly presented in the findings? | Yes |
| 32. Clarity of minor themes | Is there a description of diverse cases or discussion of minor themes? | Yes |

**Table S2. Themes and Sub-themes Discerned from Qualitative Interviews**

| **Theme** | **Sub-theme** | **Core Description** | **Representative Quote** |
| --- | --- | --- | --- |
| 1. Knowledge Acquisition and Application Gaps | 1.1 Impactful and Practical Knowledge | Knowledge points highly relevant to clinical work, brilliantly delivered or with vivid case studies. | *"What left the deepest impression on me was the rational clinical use of anti-tumor drugs and the key review points for proton pump inhibitors. These two left a strong impression." (T4) "Teacher Jia Xuedong's lecture covered how adverse drug reactions can lead to medication errors, which in turn may worsen a patient's condition. I don't usually focus on this aspect, so it left a strong impression on me."(T12)* |
|  | 1.2 Disconnect Between Learning and Application/ Contextual Mismatch | Difficulty applying learned knowledge due to differences in hospital drug formularies, lack of systems, or specialty misalignment. | *"Because our hospital doesn't have anti-tumor drugs, I listened more carefully to that part." (T4) "Since our institution is a maternal and child health hospital, we would naturally hope to be assigned to a corresponding institution of the same specialty for training." (T4)* |
|  | 1.3 Uncovered Practical Difficulties | Challenging problems encountered in work that the training did not adequately cover or provide solutions for. | *"Given the high number of elderly patients with polypharmacy at our institution, I feel the training didn't delve deeply enough into the clinical details—specifically, how to balance therapeutic benefits with adverse reaction risks when using multiple chronic disease medications. I understand this might be due to time constraints, but I genuinely wanted to learn more about these critical details."*(T5) "*I missed the module on rule-setting at the review center, so I didn't get to learn about it*." (T4) |
| 2. Attitudinal and Perceptual Shifts | 2.1 Role Perception: From "Dispenser" to "Gatekeeper" | A fundamental shift in the perception of the prescription review role, with enhanced sense of responsibility and value. | *"Pharmacists are the first responsible persons for medication safety. In practice, since most patients at our institution take their medications and return directly home after dispensing, we truly become the final safety checkpoint in their clinical treatment. This means that during the dispensing process, we must not only verify the accuracy of medications but also consider patients' individual differences. This realization makes me feel a greater sense of responsibility, but at the same time, a stronger sense of value in what I provide to patients."* (T5) *"Through my gradual, day-to-day experiences and participation in successive training sessions, I've come to truly appreciate how essential pharmacists are. We really occupy such a central role—we can communicate directly with patients while also serving as the vital link to physicians." (T2)* |
|  | 2.2 Boost in Confidence and Self-Efficacy | Significant increase in confidence in performing review duties post-training, often quantified with scores. | *"On a 5-point scale, I would rate myself as a 3 before the training and a 5 after its completion."* (T4) "*On a 5-point scale, I'd rate myself a 2 before the training and a 4 after.*" (T11) |
|  | 2.3 Definition of an Ideal Review Pharmacist's Competencies | Recognition of the importance of comprehensive qualities beyond professional knowledge, such as communication, learning ability, and sense of responsibility. | *"First, you need to have a solid professional foundation. Then, you must have the motivation to keep learning continuously. And you also need communication and collaboration skills, as well as a strong sense of responsibility—you have to have all of these. When reviewing prescriptions, you can't say, 'Oh, it's just a minor issue, ignore it.' When it comes to patient safety, nothing is trivial. So our sense of responsibility must be extremely rigorous—we must have that rigorous sense of responsibility."*(T12) *"First, you need a solid knowledge base. Second, the ability to use new tools—before training, I didn't know where to look things up; now I know multiple channels like Wanfang Data. Even more important is clinical thinking. And communication skills—attitude also matters, whether with physicians or patients. In every profession today, service attitude is essential." (T6)* |
| 3. Behavioral Changes and Practice Barriers | 3.1 Optimization of Review Processes and Habits | Adoption of more meticulous and proactive review steps in actual work. | *"Now, however, I take a more careful approach. I ask patients, 'Have you used this drug before—like amoxicillin or cephalosporins—and did you have any allergic reactions?' For high-risk medications such as antidiabetics and anticoagulants, I specifically mark them, ask about any relevant medical conditions or concomitant drugs, and check for potential drug-drug interactions.'"* (T5) "*Now when reviewing prescriptions, I no longer intervene mechanically. Instead, I communicate with the physician and review the patient's medical records and clinical condition—not just relying on system alerts about interactions or inappropriate prescriptions.*" (T1) |
|  | 3.2 Attempted Application of New Knowledge and Tools | Efforts to apply learned concepts (e.g., tiered management, rule-setting approaches) in local practice. | *"What impressed me most was the lecture on tiered management of off-label drug use. Teacher Xiao Xu shared how their hospital implements this: evidence-supported off-label use gets a general alert and dual signatures; unsupported use gets intercepted. Our hospital only uses dual signatures now, but after returning I've suggested to leadership that we consider adopting tiered management."* (T7) "*I understand this knowledge point, but when I returned to my hospital and actually started setting up the rules myself, I still found it somewhat difficult.*" (T7) |
|  | 3.3 Major Barriers in Practice | Core challenges hindering the translation of knowledge into practice, mainly stemming from platform and interpersonal dimensions. | *"Communication skills really need practice—especially when talking with physicians. Sometimes when you reject a prescription and point out problems, some doctors just say, 'I've always prescribed it this way."* (T9) "*we still haven't implemented that pre-review platform. I guess it comes down to the hospital's budget—it's a matter of money.*" (T8) |
|  | 3.4 Internal Knowledge Sharing | Sharing of training learnings within the department in various forms, promoting collective team improvement. | *"I have shared clinical cases during morning meetings, focusing on anti-tumor therapies and their combination regimens. Additionally, I disseminate newly acquired knowledge that may be unfamiliar to colleagues. The feedback has been positive, as the shared information addressed gaps in collective understanding."* (T5) "*I prepared a PPT and presented it within our department. The response was very positive—it actually sparked discussion among them.*" (T7) |
| 4. Training Evaluation & Future Expectations | 4.1 Satisfactory Aspects | Elements of the training program, such as curriculum, instructors, and management, that received high praise. | *"A particularly satisfying aspect of the training was my placement in the outpatient western pharmacy, where I experienced genuine care from the leadership and supervising faculty. The provision of a dedicated meeting room and computers helped create a comfortable, welcoming environment that felt like our own office."* (T4) "*A key strength of the training was the high quality of the lecture materials—the PPTs were excellently designed and the lectures were highly informative. Additionally, my supervising preceptor, Wang Jing, demonstrated exceptional dedication in guiding me throughout the placement.*" (T10) |
|  | 4.2 Dissatisfactory Aspects | Specific issues related to logistics support and schedule arrangement. | *"The main dissatisfaction was with the accommodation—it was too crude. The meal allowance could also be increased. "* (T4) "*One month is quite short. Aside from the theoretical study, I would have liked to rotate through other departments as well.*" (T10, T14) |
|  | 4.3 Optimization Suggestions for Future Training | Specific, actionable suggestions for improvement and core expectations. | Extend and Deepen:*"I hope the timeline can be extended. It's not easy to get this kind of training opportunity away from work. If I were only assigned to one department—say, outpatient pharmacy or inpatient pharmacy—staying in one place feels a bit monotonous. If possible, I'd like to observe other departments too."* (T10) Specialty Alignment: "*I hope for specialty alignment. Since we are from a maternal and child health hospital, we would like to be assigned to maternal and child health institutions.*" (T4) Strengthen Practice and Communication: "*Hope to have opportunities to go to the clinical front.*" (T4) "*Add communication skills training.*" (T9) Continuous Support: "*I feel that after the training, everyone should have opportunities for continued, sustained learning—like having a platform with question banks, videos, and case studies to learn from. Learning shouldn't just end after one month.*" (T9) |

**Table S3 Correlation Matrix of KAP Scores (n=394)**

|  | **Knowledge** | **Attitude** | **Practice** |
| --- | --- | --- | --- |
| **Knowledge** | 1.000 |  |  |
| **Attitude** | 0.781^**^(0.745–0.812) | 1.000 |  |
| **Practice** | 0.821^**^ (0.791–0.846) | 0.708^**^(0.658–0.750) | 1.000 |

Note: **P < 0.05, 95% confidence intervals in parentheses.

**Table S4. Response of study participants for Knowledge, Attitude, and Practice questions.**

| **Dimensions** | **Questions** | **N (%)** | | | | |
| --- | --- | --- | --- | --- | --- | --- |
|  |  | **Strongly Disagree** | **Disagree** | **Neutral** | **Agree** | **Strongly Agree** |
| Knowledge: The Impact of Training on Your Professional Knowledge Reserve | K1 The training content comprehensively covered the core knowledge and skills of prescription review. | 0(0.0) | 0(0.0) | 4(1.0) | 84(21.3) | 304(77.2) |
|  | K2 The training content was highly relevant to my actual work needs. | 0(0.0) | 0(0.0) | 32(8.1) | 98(24.9) | 264(67.0) |
|  | K3 The training content was well-aligned with the specialized characteristics of my hospital. | 0(0.0) | 0(0.0) | 30(7.6) | 104(26.4) | 260(66.0) |
|  | K4 The training significantly improved my professional knowledge level in prescription review. | 0(0.0) | 0(0.0) | 6(1.5) | 86(21.8) | 302(76.6) |
|  | K5 The interdisciplinary knowledge covered in the training (e.g., clinical pharmacology, diagnostics) is sufficient to support my daily review work. | 0(0.0) | 0(0.0) | 24(6.1) | 122(31.0) | 248(62.9) |
|  | K6 The typical cases studied in the training were very helpful for me in handling complex and difficult prescriptions. | 0(0.0) | 0(0.0) | 14(3.6) | 114(28.9) | 266(67.5) |
|  | K7 The training enables me to identify potential risks in prescriptions more quickly and accurately. | 0(0.0) | 0(0.0) | 10(2.5) | 92(23.4) | 292(74.1) |
| Attitude : The Impact of Training on Your Work Perception and Beliefs | A1 The training gave me a deeper understanding of the importance of the prescription review role in ensuring patient safety. | 0(0.0) | 0(0.0) | 2(0.5) | 64(16.2) | 328(83.2) |
|  | A2 The training strengthened my sense of responsibility and mission as a "gatekeeper of medication safety". | 0(0.0) | 0(0.0) | 2(0.5) | 72(18.3) | 320(81.2) |
|  | A3 The training boosted my confidence in doing prescription review work well. | 0(0.0) | 0(0.0) | 4(1.0) | 74(18.8) | 316(80.2) |
|  | A4 The training has increased my willingness to proactively participate in rational medication use interventions. | 0(0.0) | 0(0.0) | 2(0.5) | 76(19.3) | 316(80.2) |
|  | A5 The training increased my willingness and confidence to communicate effectively with physicians regarding prescription issues. | 0(0.0) | 0(0.0) | 6(1.5) | 86(21.8) | 302(76.6) |
|  | A6 The training has increased my willingness to proactively participate in rational medication use interventions. | 0(0.0) | 0(0.0) | 8(2.0) | 86(21.8) | 300(76.1) |
| Practice: The Impact of Training on Your Actual Work Behavior | P1 I have applied the standard prescription review processes and norms learned in the training to my daily work. | 0(0.0) | 0(0.0) | 22(5.6) | 104(26.4) | 268(68.0) |
|  | P2 I now intervene more proactively and promptly on prescriptions with potential risks. | 0(0.0) | 0(0.0) | 12(3.0) | 118(29.9) | 264(67.0) |
|  | P3 I habitually use the tools or methods recommended in the training (such as evidence-based databases) for information retrieval and decision-making. | 0(0.0) | 0(0.0) | 32(8.6) | 120(30.5) | 240(60.9) |
|  | P4 I try to actively share the new knowledge and methods learned in the training with my department colleagues. | 0(0.0) | 0(0.0) | 10(2.5) | 92(23.4) | 292(74.1) |
|  | P5 When faced with a doctor's query, I can explain my review opinions more professionally and systematically. | 0(0.0) | 2(0.5) | 18(4.6) | 126(32.0) | 248(62.9) |
|  | P6 After returning to my post, both the quantity and quality of prescriptions I have intervened in have improved. | 0(0.0) | 2(0.5) | 24(6.3) | 98(24.9) | 270(68.5) |
|  | P7 I will try to apply the logic/rationale for setting prescription review rules learned in the training to my work (e.g., system rule optimization, manual review criteria). | 0(0.0) | 0(0.0) | 22(5.6) | 104(26.4) | 268(68.0) |

**Table S5. Response of study participants regarding teaching evaluation and optimization expectations.**

| **Dimension** | **Item** | **N (%)** | | | | |
| --- | --- | --- | --- | --- | --- | --- |
|  |  | **Very unhelpful** | **Slightly unhelpful** | **Neutral** | **Helpful** | **Very helpful** |
| Evaluation of Teaching Method Effectiveness: Please rate the actual helpfulness of the following potential teaching methods in training for your learning outcomes. | Theoretical Lectures (systematic explanation of knowledge points) | 0(0.0) | 0(0.0) | 12(3) | 86(21.8) | 296(75.1) |
|  | Case-Based Learning (analysis of real prescription cases) | 0(0.0) | 0(0.0) | 8(2) | 88(22.3) | 298(75.6) |
|  | Practical Hands-on Operation (computer simulation / on-site prescription review) | 0(0.0) | 0(0.0) | 18(4.6) | 74(18.8) | 302(76.6) |
|  | Small Group Discussion / Problem-Based Seminar | 0(0.0) | 0(0.0) | 24(6.1) | 84(21.3) | 286(72.6) |
|  | Role-Playing (simulating doctor-pharmacist communication) | 0(0.0) | 2(0.5) | 40(10.2) | 98(24.9) | 254(64.5) |
|  | Multi-Department Rotation for On-site Observation (e.g., outpatient clinic, inpatient ward, intravenous admixture service prescription review workflow) | 0(0.0) | 2(0.5) | 34(8.6) | 78(19.8) | 280(71.1) |
| **Dimension** | **Item** | **Not at all needed** | **Not needed** | **Neutral** | **Needed** | **Very much needed** |
| Expectations for Teaching Content Optimization: Based on your actual post-training work, please rate the urgency of the following potential training content for you. | Medication Review for Special Populations (patients with hepatic/renal impairment, children, elderly, pregnant/lactating women) | 0(0.0) | 2(0.5) | 16(4.1) | 68(17.3) | 308(78.2) |
|  | Specialized Drug Management and Review (e.g., antimicrobials, antineoplastic drugs) | 0(0.0) | 2(0.5) | 20(5.1) | 72(18.3) | 300(76.1) |
|  | Safe Medication Use and Review Key Points for Newly Marketed Drugs & Novel Therapies | 2(0.5) | 4(1) | 36(9.1) | 74(18.8) | 278(70.6) |
|  | Laws, Regulations, Policies, and Ethical Issues Related to Prescription Review | 0(0.0) | 0(0.0) | 8(2) | 92(23.4) | 294(74.6) |
|  | Effective Communication Skills and Art with Physicians, Nurses, and Patients | 0(0.0) | 0(0.0) | 20(5.1) | 82(20.8) | 292(74.1) |
|  | Difficult Prescription Consultation and Multidisciplinary Team (MDT) Collaboration Skills | 0(0.0) | 6(1.5) | 18(4.6) | 88(22.3) | 282(71.6) |
|  | Operation and Rule-Setting for Prescription Pre-review Systems | 2(0.5) | 6(1.5) | 10(2.5) | 90(22.8) | 286(72.6) |
|  | Off-label Drug Use: Record-filing Process and Review Key Points | 0(0.0) | 0(0.0) | 10(2.5) | 90(22.8) | 294(74.6) |
|  | Integration of Medical Insurance Policies and Prescription Review | 0(0.0) | 0(0.0) | 16(4.1) | 82(20.8) | 296(75.1) |
| **Dimension** | **Item** | **Not at all anticipated** | **Not anticipated** | **Neutral** | **Anticipated** | **Very much anticipated** |
| Expectations for Teaching Support & Service Improvement: Please rate your level of expectation for the following training improvement measures. | Increase the number of practical clinical cases and analyses | 0(0.0) | 0(0.0) | 6(1.5) | 76(19.3) | 312(79.2) |
|  | Provide post-training online review material packages (slides, videos, guidelines) | 0(0.0) | 0(0.0) | 6(1.5) | 74(18.8) | 314(79.7) |
|  | Provide post-training continuous learning resources (e.g., case libraries, rule templates) | 0(0.0) | 0(0.0) | 6(1.5) | 74(18.8) | 314(79.7) |
|  | Establish a mentor database for long-term online consultation and guidance after returning to work | 0(0.0) | 0(0.0) | 8(2) | 70(17.8) | 316(80.2) |
|  | Establish a trainee communication platform (e.g., WeChat group) to facilitate peer support | 0(0.0) | 0(0.0) | 8(2) | 68(17.3) | 318(80.7) |
|  | Organize regular advanced training or thematic seminars | 0(0.0) | 0(0.0) | 10(2.5) | 72(18.3) | 312(79.2) |
|  | Closer integration of training assessment and job competency certification | 0(0.0) | 0(0.0) | 8(2) | 74(18.8) | 312(79.2) |
|  | Optimize living arrangements during training (e.g., accommodation, meal allowances) | 0(0.0) | 0(0.0) | 22(5.6) | 62(15.7) | 310(78.7) |

**Figure S1 Scatterplots with linear regression lines and 95% confidence intervals for pairwise correlations among knowledge, attitude, and practice scores (n=394).**

**(A) knowledge vs. practice; (B) knowledge vs. attitude; (C) attitude vs. practice.**

**Supplementary Questionnaire**

**Questionnaire on the Training Effectiveness and Demand of Returning Trainees in Prescription Review Post**

Dear Colleagues,

Greetings! We are conducting a research study on the effectiveness and optimization needs of prescription review training programs. The purpose of this study is to understand your real experiences, gains, and expectations after returning to work following the previous training. Your valuable opinions will provide crucial basis for the optimization of future training courses.

This questionnaire is filled out anonymously. All data will only be used for overall statistical analysis, and we will strictly keep your personal information confidential. It will take approximately 5-10 minutes to complete. Your participation is vital to this study, and we sincerely appreciate your support and cooperation!

1.I have read and understood the above information and voluntarily agree to participate in this study.

□YES □NO

**Part 1: Basic Information**

Notes: Please tick "√" in the box corresponding to your situation or fill in the blanks.

2. Gender: □ Male □ Female

3. Age: ______ years old

4. Educational level:

□ College Diploma □ Associate Degree □ Bachelor’s Degree □ Master’s Degree

5.Hospital grade: □ Grade III Hospital □ Grade II Hospital □ Community Health Service Center/Station □ Township Health Center □ Others (please specify): ______

6.Your Professional and Technical Title: □ Pharmacist Assistant (Junior-level Title) □ Pharmacist (Intermediate-level Title) □ Supervisor Pharmacist (Intermediate-level Title) □ Associate Chief Pharmacist (Senior-level Title) □ Chief Pharmacist (Senior-level Title)

7. Years of working experience in pharmacy-related fields: ______ years

8. Years of working experience in prescription review: ______ years

9. Total duration of this prescription review post training: □ 2 weeks □ 1 month □ 3 months □ 6 months □ 1 year □ Others (please specify): ______

10. Did you rotate to other departments during the training? □YES □NO

11. How long has it been since you completed this training (i.e., returned to work)? ______ months

12. Has your hospital implemented a prescription pre-review platform?□YES □NO

13. Your main work post after returning to work? 【Multiple choices】 □ Prescription Review □ Drug Dispensing □ Drug Storage Management □ Clinical Pharmacy □ Clinical Pharmacology □ Others

**Part 2: Training Effectiveness Evaluation Scale (KAP)**

Instructions: Please judge your degree of agreement with the following statements and tick "√" on the corresponding number on the right based on your true situation and feelings after participating in the training and returning to work.

| **Dimensions** | **Questions** | **Strongly Disagree** | **Disagree** | **Neutral** | **Agree** | **Strongly Agree** |
| --- | --- | --- | --- | --- | --- | --- |
| 14. Knowledge: The Impact of Training on Your Professional Knowledge Reserve | K1 The training content comprehensively covered the core knowledge and skills of prescription review. | □ | □ | □ | □ | □ |
|  | K2 The training content was highly relevant to my actual work needs. | □ | □ | □ | □ | □ |
|  | K3 The training content was well-aligned with the specialized characteristics of my hospital. | □ | □ | □ | □ | □ |
|  | K4 The training significantly improved my professional knowledge level in prescription review. | □ | □ | □ | □ | □ |
|  | K5 The interdisciplinary knowledge covered in the training (e.g., clinical pharmacology, diagnostics) is sufficient to support my daily review work. | □ | □ | □ | □ | □ |
|  | K6 The typical cases studied in the training were very helpful for me in handling complex and difficult prescriptions. | □ | □ | □ | □ | □ |
|  | K7 The training enables me to identify potential risks in prescriptions more quickly and accurately. | □ | □ | □ | □ | □ |
| 15. Attitude : The Impact of Training on Your Work Perception and Beliefs | A1 The training gave me a deeper understanding of the importance of the prescription review role in ensuring patient safety. | □ | □ | □ | □ | □ |
|  | A2 The training strengthened my sense of responsibility and mission as a "gatekeeper of medication safety". | □ | □ | □ | □ | □ |
|  | A3 The training boosted my confidence in doing prescription review work well. | □ | □ | □ | □ | □ |
|  | A4 The training has increased my willingness to proactively participate in rational medication use interventions. | □ | □ | □ | □ | □ |
|  | A5 The training increased my willingness and confidence to communicate effectively with physicians regarding prescription issues. | □ | □ | □ | □ | □ |
|  | A6 The training has increased my willingness to proactively participate in rational medication use interventions. | □ | □ | □ | □ | □ |
| 16. Practice: The Impact of Training on Your Actual Work Behavior | P1 I have applied the standard prescription review processes and norms learned in the training to my daily work. | □ | □ | □ | □ | □ |
|  | P2 I now intervene more proactively and promptly on prescriptions with potential risks. | □ | □ | □ | □ | □ |
|  | P3 I habitually use the tools or methods recommended in the training (such as evidence-based databases) for information retrieval and decision-making. | □ | □ | □ | □ | □ |
|  | P4 I try to actively share the new knowledge and methods learned in the training with my department colleagues. | □ | □ | □ | □ | □ |
|  | P5 When faced with a doctor's query, I can explain my review opinions more professionally and systematically. | □ | □ | □ | □ | □ |
|  | P6 After returning to my post, both the quantity and quality of prescriptions I have intervened in have improved. | □ | □ | □ | □ | □ |
|  | P7 I will try to apply the logic/rationale for setting prescription review rules learned in the training to my work (e.g., system rule optimization, manual review criteria). | □ | □ | □ | □ | □ |

**Part 3: Teaching Evaluation and Optimization Expectations**

Instructions: Please judge your degree of agreement with the following statements and tick "√" on the corresponding number on the right based on your true situation and feelings after participating in the training and returning to work.

| **Dimension** | **Item** | **Very unhelpful** | **Slightly unhelpful** | **Neutral** | **Helpful** | **Very helpful** |
| --- | --- | --- | --- | --- | --- | --- |
| 17. Evaluation of Teaching Method Effectiveness: Please rate the actual helpfulness of the following potential teaching methods in training for your learning outcomes. | Theoretical Lectures (systematic explanation of knowledge points) | □ | □ | □ | □ | □ |
|  | Case-Based Learning (analysis of real prescription cases) | □ | □ | □ | □ | □ |
|  | Practical Hands-on Operation (computer simulation / on-site prescription review) | □ | □ | □ | □ | □ |
|  | Small Group Discussion / Problem-Based Seminar | □ | □ | □ | □ | □ |
|  | Role-Playing (simulating doctor-pharmacist communication) | □ | □ | □ | □ | □ |
|  | Multi-Department Rotation for On-site Observation (e.g., outpatient clinic, inpatient ward, intravenous admixture service prescription review workflow) | □ | □ | □ | □ | □ |
| **Dimension** | **Item** | **Not at all needed** | **Not needed** | **Neutral** | **Needed** | **Very much needed** |
| 18. Expectations for Teaching Content Optimization: Based on your actual post-training work, please rate the urgency of the following potential training content for you. | Medication Review for Special Populations (patients with hepatic/renal impairment, children, elderly, pregnant/lactating women) | □ | □ | □ | □ | □ |
|  | Specialized Drug Management and Review (e.g., antimicrobials, antineoplastic drugs) | □ | □ | □ | □ | □ |
|  | Safe Medication Use and Review Key Points for Newly Marketed Drugs & Novel Therapies | □ | □ | □ | □ | □ |
|  | Laws, Regulations, Policies, and Ethical Issues Related to Prescription Review | □ | □ | □ | □ | □ |
|  | Effective Communication Skills and Art with Physicians, Nurses, and Patients | □ | □ | □ | □ | □ |
|  | Difficult Prescription Consultation and Multidisciplinary Team (MDT) Collaboration Skills | □ | □ | □ | □ | □ |
|  | Operation and Rule-Setting for Prescription Pre-review Systems | □ | □ | □ | □ | □ |
|  | Off-label Drug Use: Record-filing Process and Review Key Points | □ | □ | □ | □ | □ |
|  | Integration of Medical Insurance Policies and Prescription Review | □ | □ | □ | □ | □ |
| **Dimension** | **Item** | **Not at all anticipated** | **Not anticipated** | **Neutral** | **Anticipated** | **Very much anticipated** |
| 19. Expectations for Teaching Support & Service Improvement: Please rate your level of expectation for the following training improvement measures. | Increase the number of practical clinical cases and analyses | □ | □ | □ | □ | □ |
|  | Provide post-training online review material packages (slides, videos, guidelines) | □ | □ | □ | □ | □ |
|  | Provide post-training continuous learning resources (e.g., case libraries, rule templates) | □ | □ | □ | □ | □ |
|  | Establish a mentor database for long-term online consultation and guidance after returning to work | □ | □ | □ | □ | □ |
|  | Establish a trainee communication platform (e.g., WeChat group) to facilitate peer support | □ | □ | □ | □ | □ |
|  | Organize regular advanced training or thematic seminars | □ | □ | □ | □ | □ |
|  | Closer integration of training assessment and job competency certification | □ | □ | □ | □ | □ |
|  | Optimize living arrangements during training (e.g., accommodation, meal allowances) | □ | □ | □ | □ | □ |

**Part 4: Open-Ended Questions**

If you have any additional thoughts, please feel free to write them here.

20. What do you think is the biggest difficulty or challenge that currently prevents you from fully applying what you have learned in the training to practice? (You can explain from aspects such as the matching of knowledge with practical operations, system support, communication and collaboration, etc.): ______

21. In addition to the above content, do you have any other opinions or suggestions on the prescription review post training? (Suggestions on training duration, base selection, living security, etc., can be mentioned.): ______
